# Interventions for Addressing Anemia Among Children and Adolescents: An Overview of Systematic Reviews

**DOI:** 10.3389/fped.2020.549549

**Published:** 2021-02-16

**Authors:** Prasanna Mithra, Mahalaqua Nazli Khatib, Anju Pradhan Sinha, Nithin Kumar, Ramesh Holla, Bhaskaran Unnikrishnan, Ratheebhai Vijayamma, N. Sreekumaran Nair, Abhay Gaidhane, Syed Quazi Zahiruddin

**Affiliations:** ^1^Department of Community Medicine, Kasturba Medical College Mangalore, Manipal Academy of Higher Education, Manipal, India; ^2^Division of Evidence Synthesis, Jawaharlal Nehru Medical College, Datta Meghe Institute of Medical Sciences, Wardha, India; ^3^Division of Reproductive, Maternal and Child Health, Indian Council of Medical Research, New Delhi, India; ^4^Manipal Institute of Communication, Manipal Academy of Higher Education, Manipal, India; ^5^Medical Biometrics & Informatics (Biostatistics), Jawaharlal Institute of Postgraduate Medical Education and Research, Puducherry, India; ^6^Department of Community Medicine, School of Epidemiology and Public Health, Jawaharlal Nehru Medical College, Datta Meghe Institute of Medical Sciences, Wardha, India

**Keywords:** childhood anemia, adolescent anemia, supplementation, fortification, overview of review

## Abstract

**Context:** Anemia is a public health problem that can lead to growth, cognitive, and motor impairments.

**Objective:** To collate evidence on interventions for addressing childhood and adolescent anemia.

**Methods:** In this overview of systematic reviews, we included Cochrane as well as non-Cochrane systematic reviews (SRs) irrespective of language and publication status. Two sets of review authors independently screened articles for eligibility and extracted data from relevant SRs. We present data in a tabular format and summarize results based on outcome reported, age of participants, and type of interventions. We also adopt a “measurement for change” approach to assess the utility of measurement for development of interventions in childhood and adolescent anemia.

**Results:** Our search yielded 2,601 records of which 31 SRs were found eligible for inclusion. Results were favorable for fortification and supplementation with clear reduction in the risk of anemia and increase in hemoglobin levels across all age groups. Other interventions reported by the SRs were inconclusive and suggest further research.

**Conclusions:** Current evidence suggests that fortification or supplementation with iron and micronutrients leads to better reduction in the risk of anemia and improvements in hemoglobin levels among children and adolescents. Results of this overview can help decision makers in informing selection of interventions to address childhood and adolescent anemia.

**Review Registration:** PROSPERO CRD42016053687.

## Introduction

Anemia is a condition in which there is either a decrease in the number of red blood corpuscles (RBCs) or a decrease in the total amount of hemoglobin (Hb) (1). The World Health Organization (WHO) defines childhood anemia as Hb concentration below 11 grams per deciliter in children between 6 and 59 months, below 11.5 grams per deciliter in children between 5 and 11 years, and below 12 grams per deciliter in children between 12 and 14 years of age (2).

The highest burden of anemia is in low- and middle-income countries (LMICs). Estimates show that almost 90,000 deaths have occurred due to iron deficiency anemia across all age groups (3). India, with ~89 million children with anemia is the highest contributor of childhood anemia (4, 5). Iron deficiency anemia is the most common cause of anemia on a global level. Other causes of childhood anemia include nutritional deficiencies, such as deficiency of folic acid, iron, or vitamin B_12_; chronic conditions, such as inflammatory disorders; and parasitic infestations (2). Disorders associated with synthesis of hemoglobin, formation of RBCs, or survival of RBCs are the other, less common causes of anemia in children (2). Malaria, HIV, tuberculosis, and helminthic infestations have been reported to lead to substantial burden of anemia, especially in developing countries (2, 6, 7). The severity of anemia is higher in children under 8 years old as compared with children over 8 years (8). Regardless of the etiology of anemia, impaired physical growth, motor development, and cognitive development have been observed in anemic children (7, 9–12).

WHO and the United Nations International Children's Emergency Fund (UNICEF) recommend multipronged public health preventive strategies, such as supplementation of nutrients, fortification of food with nutrients, educational interventions, and prevention and control of parasitic and protozoal infestations (13).

Our comprehensive search strategy reveals many systematic reviews (SRs) that assess the efficacy of different public health interventions for addressing childhood anemia. However, no synthesis of these reviews has been reported in an overview. Hence, the Indian Council of Medical Research (ICMR) task force on childhood and adolescent anemia commissioned this overview in order to collate evidence on the efficacy of various interventions in addressing childhood and adolescent anemia in order to inform programs and a future research agenda. This review could serve as a baseline for clinicians, stakeholders, and decision makers to intervene in childhood and adolescent anemia.

## Methods

A letter of intent was invited from Indian scientists with experience in evidence synthesis. A review team of experienced researchers was constituted to work on the overview of SRs. The protocol was registered in the PROSPERO prospective register of systematic reviews (Registration number: CRD42016053687) after consultation with national experts (14).

### Inclusion Criteria

Irrespective of language and publication status, we included SRs, evaluating the effect of

1. Any one intervention, or
2. Combination of interventions, such as iron of ferrous sulfate (FS) or zinc (Zn) or multi micro nutrients (MMN) or vitamin supplementation, or
3. Iron or FS or Zn or MMN or vitamin fortification, or
4. Anti-helminthic treatment
5. Treatment or prevention of malaria, or
6. Treatment of *H. pylori*, or
7. Water, sanitation, and hygiene (WASH) interventions.

We included SRs designed for addressing anemia in children between 6 months and 19 years of age. We included studies regardless of gender, study setting, and severity of anemia. We excluded non-SRs, SRs targeting populations other than children and adolescents, those done on overtly diseased children or adolescents, those providing data on overlapping age groups, and SRs that did not assess the outcomes of interest of this overview. We assessed outcomes, namely anemia, Hb levels, and adverse events.

### Search Methods for Identification of Reviews

We searched the electronic sources listed below between February 8, 2018, and February 10, 2018, and further updated searches between January 5, 2019, and February 7, 2019: Cochrane Database for Systematic Reviews, MEDLINE via PubMed, Database of Abstracts of Reviews of Effects, Cumulative Index to Nursing and Allied Health Literature (CINAHL), Excerpta Medica dataBASE (EMBASE), Campbell Collaboration Online Library of Systematic Reviews (www.campbellcollaboration.org/library.html), Database of Promoting Health Effectiveness Reviews (https://eppi.ioe.ac.uk/webdatabases4/Intro.aspx?ID=9), and 3ie Database of Systematic Reviews. The review team, in consultation with an information specialist (RV), decided potential key words to be used for this evidence summary. We developed the search strategy for CENTRAL as in Supplementary File 1. In addition, we hand-searched the bibliographies of all eligible reviews, pertinent clinical guidelines, and textbooks to find relevant reviews. We also communicated and requested specialists in this field to provide related unpublished work.

### Selection of Reviews

We undertook screening of SRs using Covidence SR software (15). Two overview authors (PM and RH) independently screened all SRs (retrieved in the search process) on the basis of title and abstract. Two overview authors (RH and NK) then independently screened the full texts of identified reviews for inclusion. A third reviewer (BU) resolved disagreement among primary reviewers through discussion. We prepared a PRISMA flow diagram to map the inclusion and exclusion of SRs (16).

### Data Extraction

We developed and pilot-tested our data extraction form for its suitability and usability. Three review authors (AS, PM, and NK) independently extracted data regarding characteristics of the included participants, interventions, comparisons, outcomes, and methodological quality of the included reviews. We contacted reviewers to seek missing information if any.

### Methodological Quality of Included Reviews Summarized in the Overview

Two review authors (AS and PM) independently evaluated the methodological quality of included reviews with the Revised Assessment of Multiple Systematic Reviews (R-AMSTAR) tool (17, 18).

### Data Synthesis

We followed standard procedures given by the Cochrane Handbook for Systematic Reviews of Interventions (19). We obtained data from included SRs and presented them in a tabular and graphical format. In case of duplicate publications, we considered them as a single report. We summarized results based on outcome reported, age of participants, and type of interventions as risk ratio (RR), mean difference (MD), standardized mean difference (SMD), or weighted mean difference (WMD) with 95% confidence intervals (95% CI). As an overview, we mainly focused on summarizing and thematically categorizing the intervention and its effectiveness and not taking a more interpretative approach during synthesis.

## Results

### Search Results

**Figure 1:**
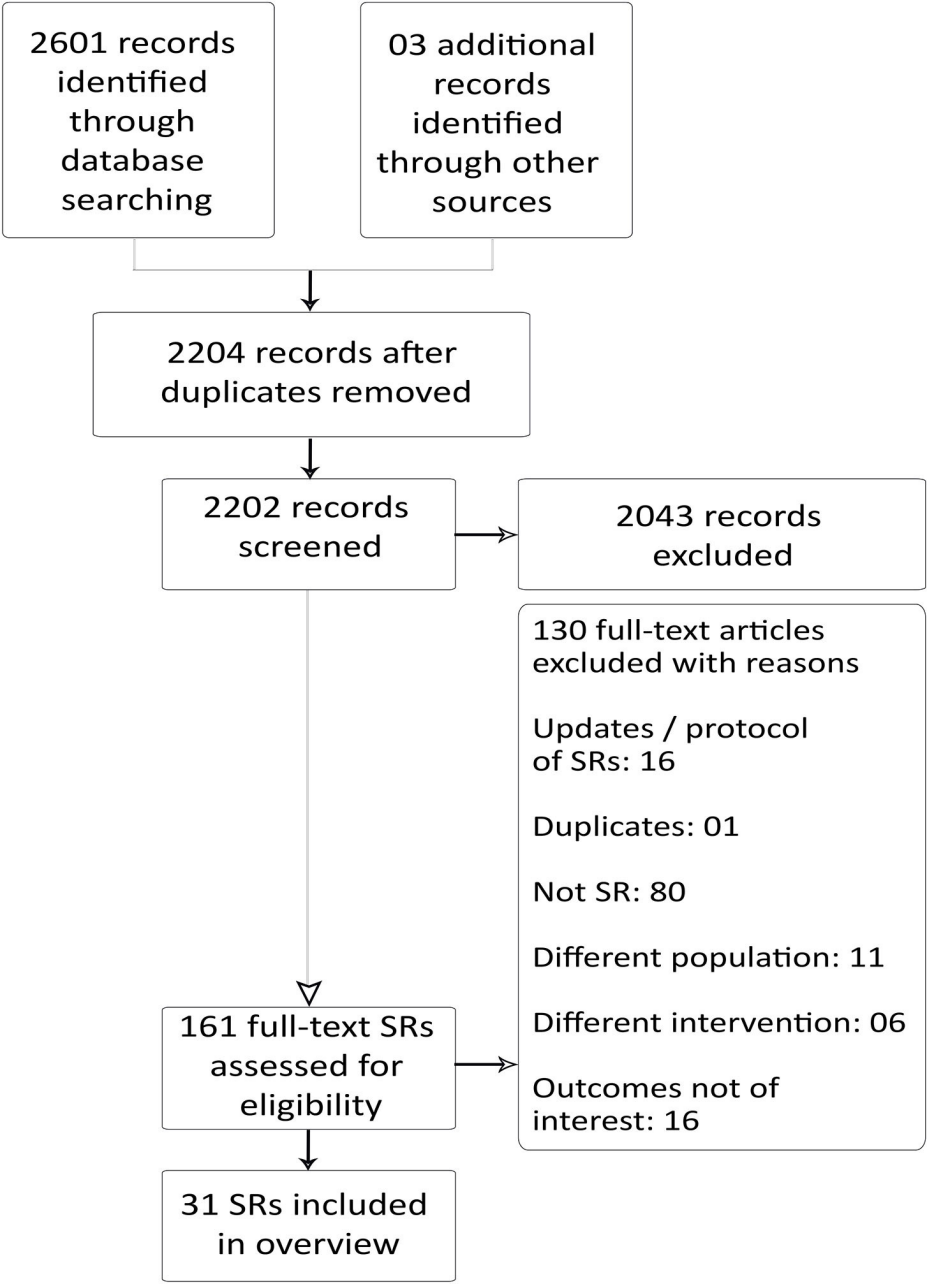
Preferred reporting items for SR and meta-analysis: the PRISMA flow diagram for inclusion and exclusion of studies.

Our search yielded 2,601 records. After removal of duplicates, 2,204 records remained, and they were screened for title and abstract. At this stage, we excluded 2,043 articles, and a total of 161 full-text articles were screened for eligibility. We excluded 130 articles and included 31 SRs that met our inclusion criteria (Figure 1).

### Description of Included Reviews

We included 31 SRs in this overview, the salient features of which are depicted in Table 1.

**Table 1:** Characteristics of the included systematic reviews.

**SN**	**Study ID**	**No of trials/Sample size**	**Participants**	**Intervention**	**Outcomes reported**
**SUPPLEMENTATION**					
1.	Low et al. (20)	10–50 years age: 67/8,506 12–18 years age: 10/3,220	Menstruating women aged 10–50 years Subgroup analysis done for 12–18 years	Daily oral iron supplementation with or without other vitamins (folic acid or vitamin C)	Anemia Hemoglobin Biomarkers (Ferritin)
2.	Neuberger et al. (21)	35/31,955	<18 years children in malaria-endemic areas ± anemia ± malaria	Iron, iron + folic acid, and iron + antimalarial	Anemia Hemoglobin Adverse effects
3.	Mayo-Wilson et al. (22)	80/2,05,401	6 months to 12 years of age	Orally administered zinc supplementation and non-zinc co-interventions (e.g., vitamin A, additional iron)	Anemia Hemoglobin Adverse effects Biomarkers (Fe)
4.	Cembranel et al. (23)	13/1,699	Children <5 years of age	Weekly/daily supplementation of iron	Anemia Hemoglobin
5.	Low et al. (24)	32/7,089	Children between 3.3 and 15 years of age	Iron supplementation	Anemia Hemoglobin Adverse effects Biomarkers (Ferritin)
6.	Pasricha et al. (25)	33/42,015	4–23 months of age	Daily iron supplementation	Anemia Hemoglobin Adverse effects Biomarkers (Ferritin, iron, transferrin, erythrocyte protoporphyrin)
7.	Thompson et al. (26)	15/2,154	2–5 years	Daily oral iron supplementation	Hemoglobin Biomarkers (Ferritin)
8.	Abdullah et al. (27)	2/69	6–30 months	Oral ferrous glycine sulfate and ferrous sulfate	MA not done due to high heterogeneity
9.	De-Regil et al. (28)	33/13,114	Apparently healthy children <12 years	Intermittent oral iron supplementation alone or in combination with other vitamins and minerals	Anemia Hemoglobin Adverse effects Biomarkers (Ferritin)
10.	Ramakrishnan et al. (29)	21/4,651	6 month to 18 years	Iron interventions	Hemoglobin
**FORTIFICATION**					
11.	Matsuyama et al. (30)	15 articles based on 12 studies/NR	Healthy children aged 6–47 months	Fortified milk	Anemia Hemoglobin Biomarkers (Serum Ferritin, serum transferrin receptor concentration)
12.	De-Regil et al. (31)	13 trials (17 reports)/5,810	Preschool and school-age children from Latin America, Africa, and Asia	Point-of-use fortification of foods with MNP	Anemia Hemoglobin Adverse effects Biomarkers (Ferritin)
13.	Aaron et al. (32)	10	50525 apparently healthy children and women of reproductive age from LMICs	Non-dairy MMN fortified beverages	Anemia
14.	Das et al. (33)	11	Children and women (presented separate data for children) <11 year old children, pre-term infants, malnourished infants, and school children with asymptomatic zinc deficiency)	Zinc fortified formula feeds	Hemoglobin
15.	Das et al. (34)	201 (121 on infants and children)	Children <18 years of age and women (presented separate data for infants and children)	Vitamin A, iron and multiple micronutrients fortified formula food	Anemia Hemoglobin Biomarkers (Ferritin)
16.	Salam et al. (35)	17	Women and children (6–66 months of age) (no studies identified on women)	Point-of-use powders with ≥2 micronutrients in their formulation (Vit A, C, B 11, D, B complex, Fe, Zinc, lysine)	Anemia Hemoglobin Biomarkers (Ferritin)
17.	Eichler et al. (36)	18/5,468	6 months to 5 years	Micro-nutrient fortified milk or cereal food	Anemia Hemoglobin Biomarkers (Ferritin)
18.	Gera et al. (37)	60/20,827	Apparently healthy individuals, irrespective of age. Reported separate data for children on Hb	Iron-fortified foods	Hemoglobin
19.	De-Regil et al. (38)			Home fortification with multiple micronutrient powders	
20.	Best et al. (39)	12/6,145	Children between 5.5 and 18 years	Minimum 3 micronutrients added to beverages, foods	Not done
21.	Dewey et al. (40)	16/6,113	Children between 6 months and 2 years	Sprinkles, Crushable, or chewable tablets, Lipid-based nutrient supplements, soya based products.	Anemia Hemoglobin Adverse events Biomarkers (Ferritin)
**SUPPLEMENTATION WITH FORTIFICATION**					
22.	Kristjansson et al. (41)	32	3 months to 5 years socio-economically disadvantaged LMIC and HICs	Macronutrient supplementation/fortification	Anemia Hemoglobin
23.	McDonagh et al. (42)	10/5,671	Children aged between 6 and 24 months from developed countries	Oral iron supplementation, iron fortified formula, and food	MA not done
24.	Gera et al. (43)	30/6,464	Birth to 15 years	Iron and multiple micronutrient supplementation ± fortification	Hemoglobin
25.	Gera et al. (44)	55/27,945	<18 years of age	Iron supplementation, formula milk, fortified cereals	Hemoglobin
26.	Sun et al. (45)	6/676	Chinese children between 6 months and 13 years with iron deficiency anemia in	Dietary interventions	Anemia
**DEWORMING**					
27.	Taylor-Robinson et al. (46)	45	Children aged ≤ 16 years in areas endemic for intestinal helminthes	Deworming	Hemoglobin
28.	Girum et al. (47)	8	10,05,239 school children from Asia and Africa	Deworming	Hemoglobin
***H. PYLORI*** **TREATMENT**					
29.	Huang et al. (48)	8/450	2–76 years (subgroup analysis available for children)	*H. pylori* treatment	Hemoglobin Biomarkers (Ferritin)
**WASH INTERVENTION**					
30.	Dangour et al. (49)	14/22,241	<18 years	WASH intervention	Hemoglobin (No MA) Biomarkers (Ferritin) (No MA)
**ANTI-MALARIA INTERVENTION**					
31.	Athuman et al. (50)	6/3847	Anemic children between 2 months and 9 years residing in endemic areas	Intermittent preventive antimalarial treatment	Anemia

We included nine Cochrane reviews (20, 21, 28, 31, 38, 41, 46, 49, 50) and 22 non-Cochrane SRs (22–27, 29, 30, 32–37, 39, 40, 42–45, 47, 48). Except for Cembranel et al. (23), all other reviews are in the English language. Updating the version of existing SRs was reported only by Cochrane reviews. The review by Neuberger et al. (21) is the updated version of previous publications (51, 52). Taylor-Robinson et al. (46) is updated from a previous five Cochrane reviews (53–55). One Cochrane review by De-Regil et al. (38) has been reprinted in a Cochrane review journal (56), and hence, to avoid duplication, we considered both reviews as a single report (38). Abdullah et al. (27), Best et al. (39), McDonagh et al. (42), and Dangour et al. (49) did not undertake meta-analysis.

Included reviews differed in the age groups of participants: 11 reviews included participants <5 years of age (23, 25–27, 30, 33, 35, 36, 38, 40, 41). Low et al. (20), Das et al. (34), and Huang et al. (48) included participants of age groups beyond the inclusion range in our overview, but they reported separate data for children as a subgroup analysis and, hence, were included. Aaron et al. (32) included a trial on pregnant women that was not included in the meta-analysis of anemia. Another review (35) considered included women but did not identify studies on women and, hence, was included in this overview. Except for Low et al. (24), all included reviews included participants of both genders. All SRs except McDonagh et al. (42) included studies from LMICs and developing countries. Sun et al. (45) included specifically the Chinese population. One SR included studies each on children from malaria endemic areas (21), areas endemic for intestinal helminths (46), and areas endemic for anemia (50).

Reviews covered an array of interventions for prevention, control, and/or treatment of anemia in children and adolescents. We found 11 reviews on iron supplementation (20–29, 44). Six reviews were on fortification (28, 30, 33, 35, 39, 57); five reviews on supplementation with fortification (41–45); two reviews on deworming (46, 47); and one review each on *H. pylori* treatment (48), WASH intervention (49), and antimalarial intervention (50).

### Effects of Interventions

#### Anemia

**Figure 2:**
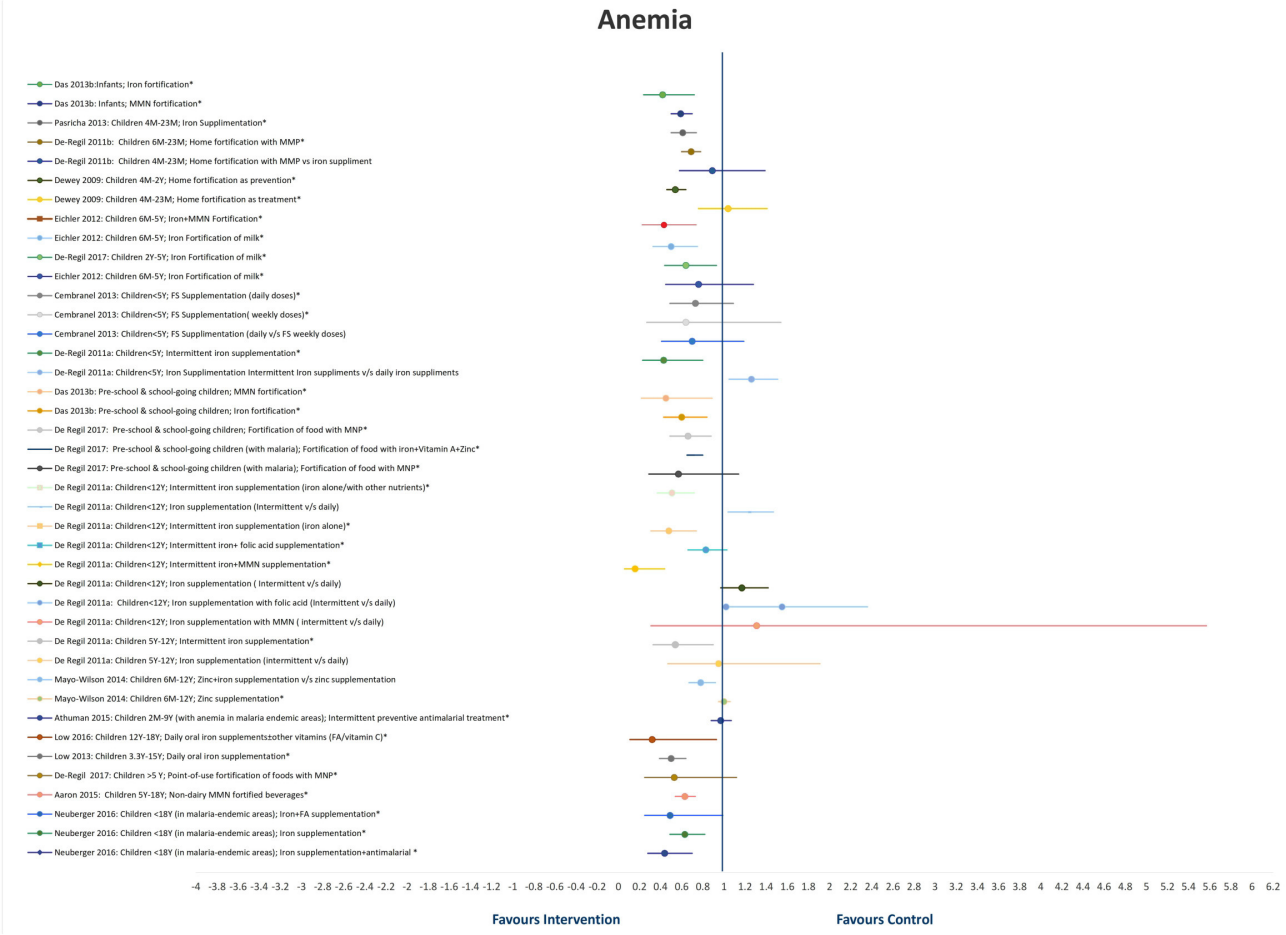
Findings of systematic reviews on anemia.

A total of 18 SRs addressed the effect of different interventions on anemia in children and adolescents (20–25, 28, 30–36, 40, 41, 45, 50) (Table 2 and Figure 2). We did not find any review that assessed the effect of treatment of deworming, *H. pylori* infection, or WASH intervention on anemia status.

**Table 2:** Findings for anemia in included systematic reviews.

**SR**		**#Included studies**	**#Participants**	**Comparison**	**Meta-analysis**	**Quality of evidence**
**INFANTS**					
Das et al. (34)	Iron fortification	6	1,234 infants	Iron fortification verses unfortified foods/ regular diet	RR (95%CI) = 0.42 (0.24, 0.72)	Moderate
Das et al. (34)	MMN fortification	3	1,809 infants	MMN fortification verses unfortified foods/ regular diet	RR (95%CI) = 0.59 (0.50, 0.70)	Low
Matsuyama et al. (30)	Fortification	NR	Healthy children <1 year of age	Fortified milk verses control	OR (95%CI) = 0.46 (0.19, 1.12)	NR
** <2 YEARS**					
Pasricha et al. (25)	Iron supplementation	17	4,825 children between 4 and 23 months	Iron supplementation verses control	RR (95%CI) = 0.61 (0.50, 0.74) *P* < 0.0001 *I*^2^ = 86%	NR
De-Regil et al. (38)	Fortification	6	1,447 children 6–23 months	Home fortification with MMP verses placebo/no intervention	RR (M-H, RE, 95%CI) = 0.69 (0.60–0.78), *I*^2^ = 19%	Moderate
De-Regil et al. (38)	Fortification	1	145 children 6–23 months	Home fortification with multiple micronutrient powders verses iron supplements	RR (M-H, RE, 95%CI) = 0.89 (0.58–1.39)	Low
Dewey et al. (40)	Fortification	–	4,331 children 6 months to 2 years	Home fortification as prevention verses control	RR (95%CI) = 0.54 (0.46, 0.64)	NR
Dewey et al. (40)	Fortification	–	1,263 children 6 months to 2 years	Home fortification as treatment verses control	RR (95%CI) = 1.04 (0.76, 1.41)	NR
** <5 YEARS**					
Matsuyama et al. (30)	Fortification	NR	Healthy children aged 6–47 months from all countries	Fortified milk verses control	OR (95%CI) = 0.32 (0.15, 0.66)	NR
Matsuyama et al. (30)	Fortification	NR	Healthy children aged 6–47 months from developing economies	Fortified milk verses control	OR (95%CI) = 0.36 (0.14, 0.91)	NR
Eichler et al. (36)	Iron + MMN fortification	7	1,927 children between 6 months and 5 years	Iron + MMN fortification verses non-fortified food	RR (95%CI) = 0.43 (0.26–0.71) *P* = 0.00 *I*^2^ = 80.5%	NR
Eichler et al. (36)	Iron fortification	11	3,100 children from 6 months to 5 years of age	Iron fortification of milk and cereals verses non-fortified food	RR (95%CI) = 0.50 (0.33, 0.75) *I*^2^ = 71.2%	NR
De-Regil et al. (31)	MNP fortification	6	1,706 children aged 24–59 months	Point-of-use fortification of foods with MNP verses no intervention or placebo	RR (M-H, RE, 95%CI) = 0.64 (0.44, 0.93) *P* = 0.019 *I*^2^ = 73%	NR
Eichler et al. (36)	Iron fortification	4	1,173 Children between 6 months and 5 years	Iron single-fortification verses control	RR (95%CI) = 0.76 (0.45–1.28) *P* = 0.533 *I*^2^ = 0%	NR
Kristjansson et al. (41)	Supplementation and fortification	1 before-after studies	110 children between 3 months and 5 years of age	Supplementary feeding verses control	OR (95%CI) = 0.58 (0.24, 0.75)	NR
Cembranel et al. (23)	FS supplementation	–	Children <5 years	Daily doses of FS verses control	RR (95%CI) = 0.73 (0.49, 1.09), *P* = 0.13, *I*^2^ > 75%	NR
Cembranel et al. (23)	FS supplementation	–	Children <5 years	Weekly doses of FS verses control	RR (95%CI) = 0.64 (0.27, 1.54), *P* = 0.33, *I*^2^ > 75%	NR
Cembranel et al. (23)	FS supplementation	–	Children <5 years	Daily doses of FS verses weekly doses	RR (95%CI) = 0.70 (0.41, 1.19), *P* = 0.09	NR
De-Regil et al. (28)	Iron supplementation	4	658 apparently healthy children <5 years	Intermittent iron supplements verses placebo	RR (M-H, RE, 95% CI) = 0.43 (0.23, 0.80)	NR
De-Regil et al. (28)	Iron supplementation	3	770 apparently healthy children <5 years	Intermittent iron supplements verses daily iron supplements	RR (M-H, RE, 95% CI) = 1.26 (1.05, 1.51)	NR
**PRE-SCHOOL AND SCHOOL-GOING CHILDREN**					
Das et al. (34)	MMN Fortification	5	1,246 pre-school and school-going children	MMN fortification verses unfortified foods/regular diet	RR (95%CI) = 0.45 (0.22, 0.89)	Low
Das et al. (34)	Iron Fortification	10	2,013 pre-school and school-going children	Iron fortification verses unfortified foods/ regular diet	RR (95%CI) = 0.60 (0.43, 0.84)	Moderate
De-Regil et al. (31)	Fortification	10	2,448 pre-school and school-age children	Point-of-use fortification of foods with MNP verses no intervention or placebo	RR (M-H, RE, 95%CI) = 0.66 (0.49–0.88) *P* = 0.004 *I*^2^ = 73%	Moderate
De-Regil et al. (31)	Fortification	7	1,705 pre-school and school-age children with malaria	Point-of-use fortification of foods with iron+ vitamin A + zinc verses no intervention or placebo	RR (M-H, RE, 95%CI) = 0.72 (0.65, 0.80) *P* = 0.47 *I*^2^ = 0%	NR
De-Regil et al. (31)	Fortification	4	934 pre-school and school-age children with malaria	Point-of-use fortification of foods with MNP verses no intervention or placebo	RR (M-H, RE, 95%CI) = 0.57 (0.29, 1.14) *P* = 0.0005 *I*^2^ = 83%	NR
** <12 YEARS**					
De-Regil et al. (28)	Iron Supplementation	10	1,824 apparently healthy children <12 years	Intermittent iron supplements iron alone or with other nutrients verses placebo or no intervention	RR (M-H, RE, 95% CI) = 0.51 (0.37, 0.72)	Moderate
De-Regil et al. (28)	Iron Supplementation	6	980 apparently healthy children <12 years	Intermittent iron supplements ± other micronutrients verses daily iron supplements ± other micronutrients	RR (M-H, RE, 95% CI) = 1.23 (1.04, 1.47) *P* = 0.017 *I*^2^ = 0%	Low
De-Regil et al. (28)	Iron Supplementation	5	1,456 apparently healthy children <12 years	Intermittent iron supplements (0–3 months duration) verses placebo or no intervention	RR (M-H, RE, 95% CI) = 0.63 (0.49, 0.82)	NR
De-Regil et al. (28)	Iron Supplementation	5	368 apparently healthy children <12 years	Intermittent iron supplements (>3 months duration) verses placebo or no intervention	RR (M-H, RE, 95% CI) = 0.37 (0.14, 1.02)	NR
De-Regil et al. (28)	Iron Supplementation	6	1074 apparently healthy children <12 years	Intermittent iron alone supplements verses placebo or no intervention	RR (M-H, RE, 95% CI) = 0.48 (0.31, 0.74)	NR
De-Regil et al. (28)	Iron Supplementation	2	593 apparently healthy children <12 years	Intermittent iron with folic acid supplements verses placebo or no intervention	RR (M-H, RE, 95% CI) = 0.83 (0.66, 1.03)	NR
De-Regil et al. (28)	Iron Supplementation	1	50 apparently healthy children <12 years	Intermittent iron with vitamin C supplements verses placebo or no intervention	RR (M-H, RE, 95% CI) = 0.06 (0.00, 0.97)	NR
De-Regil et al. (28)	Iron Supplementation	1	107 apparently healthy children <12 years	Intermittent iron with MMN supplements verses placebo or no intervention	RR (M-H, RE, 95% CI) = 0.16 (0.06, 0.44)	NR
De-Regil et al. (28)	Iron Supplementation	2	172 apparently healthy children <12 years	Intermittent iron supplements verses daily iron supplements (0 to 3 months duration)	RR (M-H, RE, 95% CI) = 1.24 (0.55, 2.77)	NR
De-Regil et al. (28)	Iron Supplementation	4	808 apparently healthy children <12 years	Intermittent iron supplements verses daily iron supplements (> 3 months)	RR (M-H, RE, 95% CI) = 1.23 (1.03, 1.47)	NR
De-Regil et al. (28)	Iron Supplementation	4	507 apparently healthy children <12 years	Intermittent iron supplements verses daily iron supplements (Iron only)	RR (M-H, RE, 95% CI) = 1.17 (0.97, 1.42)	NR
De-Regil et al. (28)	Iron supplements	1	366 apparently healthy children <12 years	Intermittent iron supplements verses daily iron supplements (with folic acid)	RR (M-H, RE, 95% CI) = 1.55 (1.02, 2.36)	NR
De-Regil et al. (28)	Iron Supplementation	1	107 apparently healthy children <12 years	Intermittent iron supplements verses daily iron supplements (with MMN)	RR (M-H, RE, 95% CI) = 1.31 (0.31, 5.57)	NR
De-Regil et al. (28)	Iron Supplementation	6	1,166 apparently healthy children between 5 and 12 years	Intermittent iron supplements verses placebo or no intervention	RR (M-H, RE, 95% CI) = 0.54 (0.33, 0.90)	NR
De-Regil et al. (28)	Iron Supplementation	2	145 apparently healthy children between 5 and 12 years	Intermittent iron supplements verses daily iron supplements	RR (M-H, RE, 95% CI) = 0.95 (0.47, 1.91)	NR
Mayo-Wilson et al. (22)	Zn/Iron supplementation	3	482 children between 6 months and 12 years of age	Zinc with iron supplementation verses zinc alone	RR (95%CI) = 0.78 (0.67–0.92) *P* = 0.54 *I*^2^ = 0%	NR
Mayo-Wilson et al. (22) (Anemia prevalence)	Zn/Iron supplementation	13	4,287 children between 6 months and 12 years of age	Orally administered zinc supplementation verses no zinc supplementation	RR (95%CI) = 1.00 (0.95, 1.06) *P* = 0.05 *I*^2^ = 37%	NR
Athuman et al. (50)	Deworming	4	2,237 children between 2 months and 9 years with anemia in malaria endemic areas	Intermittent preventive ± antimalarial treatment verses placebo	RR (95%CI) = 0.97 (0.88–1.07) *I*^2^ = 29%	Moderate
Sun et al. (45)	Dietary interventions	6	676 Chinese children with iron deficiency anemia in	Dietary interventions verses control	OR (FE, 95%CI) = 5.03 (3.09, 8.18) *P* = 0.85 *I*^2^ = 0%	NR
** <18 YEARS**					
Low et al. (20)	Iron Supplementation	4	2,169 participants between 12 and 18 years	Daily oral iron supplements ± other vitamins (folic acid or vitamin C) verses control/placebo	RR (M-H, RE, 95%CI) = 0.32 (0.11, 0.93) *P* = 0.037 *I*^2^ = 97%	NR
Low et al. (24)	Iron supplements	7	1,763 children between 3.3 and 15 years	Daily iron supplements verses placebo/anti-helminthics/Zinc/multivitamin	RR (M-H, RE, 95%CI) = 0.50 (0.39–0.64) *P* ≤ 0.001 *I*^2^ = 85%	NR
De-Regil et al. (31)	Fortification	3	543 children ≥5 years	Point-of-use fortification of foods with MNP verses no intervention or placebo	RR (M-H, RE, 95%CI) = 0.53 (0.25, 1.12) *P* = 0.097 *I*^2^ = 81%	NR
Aaron et al. (32)	Fortification	6	2,828 apparently healthy children between 5 and 18 years of age	Non-dairy MMN beverages fortified compared to non-fortified beverages	RR (M-H, RE, 95%CI) = 0.63 (0.54, 0.73) *P* < 0.00001 *I*^2^ = 84%	Moderate
Neuberger et al. (21)	Iron supplementation	3	633 children in malaria endemic areas	Iron plus folic acid vs. placebo/no treatment	RR (M-H, RE, 95% CI) = 0.49 (0.25, 0.99), *P* = 0.14, *I*^2^ = 49%	NR
Neuberger et al. (21)	Iron supplementation	15	3,784 children <18 years in malaria endemic areas	Iron vs. placebo/no treatment	RR (M-H, RE, 95% CI) = 0.63 (0.49, 0.82), *P* ≤ 0.00001, *I*^2^ = 97%	NR
Neuberger et al. (21)	Iron supplementation	2	295 children <18 years in malaria endemic areas	Iron + antimalarial vs. placebo	RR (M-H, RE, 95% CI) = 0.44 (0.28, 0.70), *P* = 0.20, *I*^2^ =40%	NR

In infants, iron fortification decreased the risk of anemia by 58% (RR = 0.42, 95%CI = 0.24–0.72; six studies; 1,234 participants; *moderate QoE*) (34) and MMN fortification by 41% (RR = 0.59, 95%CI = 0.50–0.70; three studies; 1809 participants; *low QoE*) (34).

In children under 2 years of age, home fortification, when used as a preventive measure, showed maximum reduction in the risk of anemia by 46% (RR = 0·54, 95%CI = 0.46–0.64; 4,331 participants 6M−2Y) (40). However, when used as a treatment measure, it led to an insignificant increase in the risk of anemia by 4% (RR = 1.04, 95%CI = 0.76–1.41; 1,263 participants 6M−2Y) (40). In the same age group, iron supplementation significantly reduced the risk of anemia by 39% (RR = 0·61, 95%CI = 0.50–0.74; *I*^2^ = 86%; 17 studies; 4,825 participants 4–23M) (25), followed by home fortification with MMP (compared to placebo/no intervention) by 31% (RR = 0.69, 95%CI = 0.60–0·78; *I*^2^ = 19%; six studies; 1,447 participants 4–23M; *moderate QoE*) (38).

Milk fortification significantly reduced the odds of anemia in healthy children between 6 and 47 months old by 64% (OR = 0.36, 95%CI = 0.14–0.91) (30). Fortification of food with iron and MMN (RR = 0.43, 95%CI = 0.26–0.71; *I*^2^ = 80.5%; seven studies, 1,927 participants 6M−5Y) (36) and intermittent iron supplementation (RR = 0.43, 95% CI = 0.23–0.80; four studies; 658 apparently healthy children <5 years) (28) reduced the risk of anemia significantly by 57%, followed by fortification of milk and cereals with iron by 50% (RR = 0.50, 95%CI = 0.33–0.75; *I*^2^ = 71.2%; 11 studies; 3,100 participants 6M−5Y) (36), and point-of-use fortification of milk with MMP by 36% (RR = 0.64, 95%CI = 0.44–0.93; *I*^2^ = 73%; six studies; 1,706 participants 2–5Y) (31). Single-iron fortification of milk (RR = 0.76, 95%CI = 0.45–1.28; *I*^2^ = 0%; four studies; 1,173 participants 6M−5Y) (36), daily doses of FS (RR = 0.73, 95%CI = 0.49–1.09; *I*^2^ > 75%; participants aged <5Y) (23), and weekly doses of FS (RR = 0.64, 95%CI = 0.27–1.54; *I*^2^ > 75%; participants <5Y) (23) showed insignificant reductions in the risk of anemia by 36, 27, and 24%, respectively.

In preschool and school-age children, MMN fortification showed a maximum reduction in the risk of anemia by 55% (RR = 0.45, 95%CI = 0.22–0.89; five studies; 1,246 participants) (34), followed by iron fortification by 40% (RR = 0.60, 95%CI = 0.43–0.84; 10 studies; 2,013 participants) (34), point-of-use fortification of food with micro nutrient powders (MNP) by 34% (RR = 0.66, 95%CI = 0.49–0.88; *I*^2^ = 73%;10 studies; 2,448 participants; *moderate QoE*) (31), and point-of-use fortification of food with iron, vitamin A, and Zn by 28% (RR0·72, 95%CI = 0.65–0.80; *I*^2^ = 0%; seven studies; 1,705 participants with malaria) (31). In children with malaria, point-of-use fortification of food with MNP insignificantly reduced the risk of anemia by 43% (RR = 0.57, 95%CI = 0.29–1.14; *I*^2^ = 83%; four studies; 934 participants with malaria) (31).

In apparently healthy children under 12 years of age, maximum reduction in the risk of anemia is seen with intermittent iron and MMN supplementation by 84% (RR = 0.16, 95%CI = 0.06–0.44; one study; 107 participants <12 years) (28), followed by intermittent iron supplementation (iron alone) by 52% (RR = 0.48, 95%CI = 0.31–0.74; six studies; 1,074 participants <12 years). Daily iron/folic acid (FA)/MMN supplementation showed better results as compared with intermittent iron/FA/MMN supplementation (28).

Daily oral iron supplements with or without other vitamins (FA or vitamin C) reduced the risk of anemia in children between 12 and 18 years by 68% (RR = 0.32, 95%CI = 0.11–0.93; *I*^2^ = 97%; four studies; 2,169 participants) (20), and in children between 3.3 and 15 years reduced the risk of anemia by 50% (RR = 0.50, 95%CI = 0.39–0.64; *I*^2^ = 85%; seven studies; 1,763 participants) (24). Administration of non-dairy, MMN-fortified beverages in children between 5 and 18 years in LMICs effectively reduced the risk of anemia by 37% (RR = 0.63, 95%CI = 0.54–0.73; *I*^2^ = 17%; six studies; 2,995 participants) (32). In malaria-endemic areas, additional supplementation of iron along with antimalarial treatment reduced the risk of anemia by 56% (RR = 0.44, 95%CI = 0.28–0.70; *I*^2^ = 40%; two studies; 295 participants <18Y), followed by iron and FA supplementation by 51% (RR = 0.49, 95%CI = 0.25–0.99; *I*^2^ = 49%; three studies; 633 participants <18Y), and iron supplementation by 37% (RR = 0.63, 95%CI = 0.49–0.82; *I*^2^ = 97%; 15 studies; 3,784 participants <18Y) (21).

#### Hb Levels

**Figure 3:**
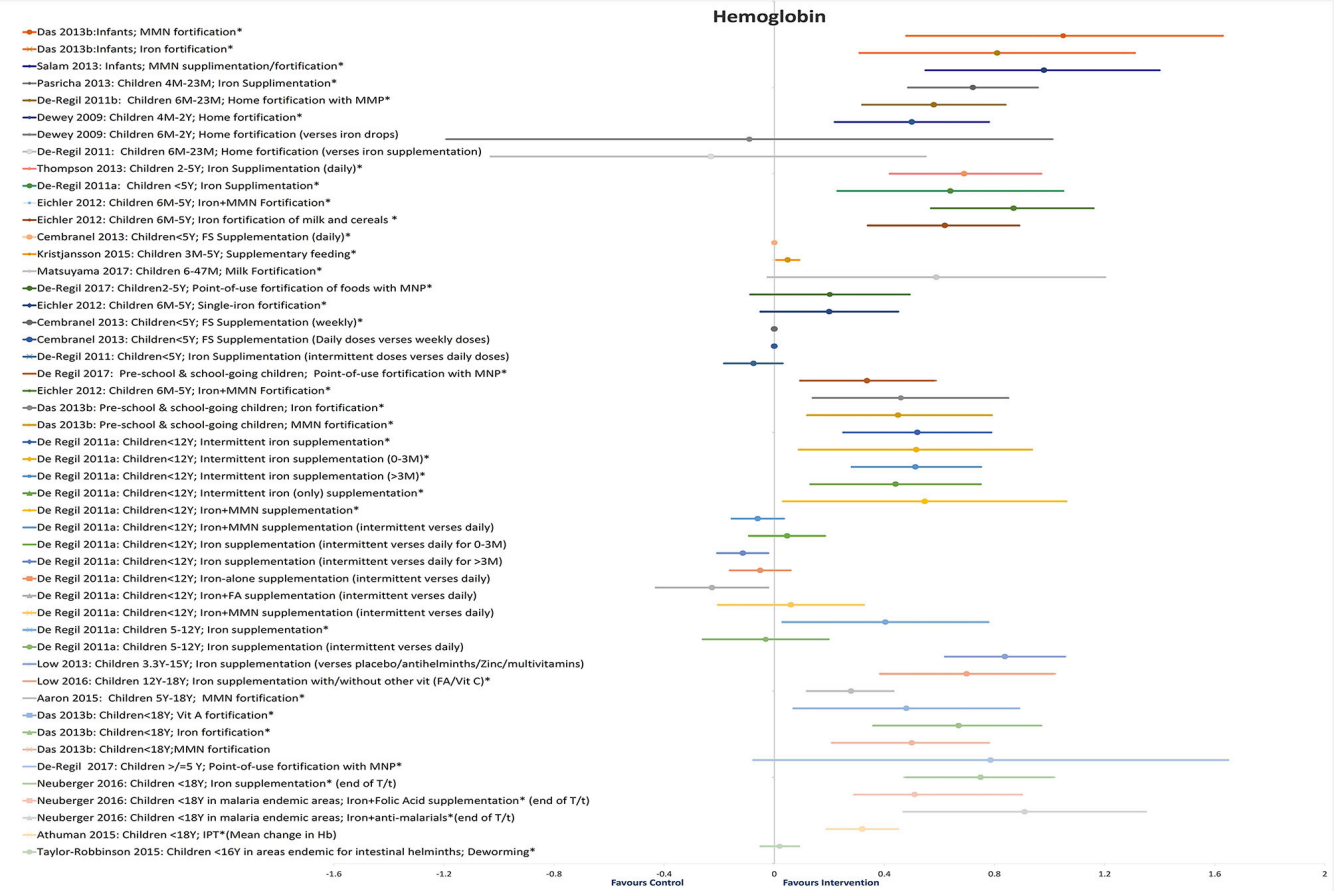
Findings of SRs on Hb.

Twenty-five reviews addressed the effect of different interventions on Hb levels in children and adolescents (20–26, 28–31, 33–38, 40, 41, 43, 44, 46–48, 50) (Table 3 and Figure 3). We did not find any review that assessed the effect of WASH intervention on Hb status.

**Table 3:** Findings for hemoglobin in included systematic reviews.

**SR**	**Intervention**	**No of included studies**	**Number of participants**	**Comparison**	**Meta-analysis**	**Quality of evidence**
**INFANTS**						
Das et al. (34)	MMN fortification	7	1,508 infants	MMN fortification verses unfortified foods/regular diet	SMD (95% CI) = 1.05 (0.48, 1.63)	Moderate
Das et al. (34)	Iron fortification	12	1,834 infants	Iron fortification verses unfortified foods/ regular diet	SMD (95% CI) = 0.81 (0.31, 1.31)	Moderate
Gera et al. (37)	Iron fortification	9	Infants	Iron fortification verses non-fortified food	WMD (95%CI) = 0.5 (0.28, 0.72) g/dL *P* ≤ 0.001 *I*^2^ = 74.7%	NR
Salam et al. (35)	MNP supplementation/fortification	14	8,354 children between 2 months and 1 year of age from developing countries	MNP vs. no intervention or control	SMD (IV, RE,95%CI) = 0.98 (0.55, 1.40) *P* < 0.00001 *I*^2^ = 99%	Moderate
Das et al. (33)	Zinc fortification	3	92 children (malnourished, preterm infants, or <9 years of age)	Zinc fortification verses control	SMD (FE, 95%CI) = −0.11(−0.52, 0.31) *P* = 0.45 *I*^2^ = 0%	NR
**CHILDREN** ** <2 YEARS OF AGE**						
Pasricha et al. (25)	Iron supplementation	26	5,479 children between 4 months and 2 years of age	Iron supplementation verses control	MD (95%CI) = 7.22 (4.87–9.57) g/L *P* < 0.0001 *I*^2^ = 94%	NR
De-Regil et al. (38)	Home fortification	6	1,447 children between 6 and 23 months	Home fortification with MMP verses placebo/no intervention	MD (IV, RE, 95%CI) = 5.87 (3.25–8.49) g/L	Moderate
Dewey et al. (40)	Home fortification	22	2,449 between children 6 months and 2 years of age	Home fortification as prevention verses control	5.06 (2.29, 7.83) g/L	NR
Gera et al. (44) (change in Hb)	Iron supplementation/ fortification	29	Children ≤ 2 years of age	Iron supplementation/ fortification verses control	WMD (RE, 95%CI) = 0.56 (0.36, 0.76) g/dL *P* < 0.001	NR
Gera et al. (37)	Iron fortification	45	Children >1 year of age	Iron fortification verses non-fortified food	WMD (RE, 95%CI) = 0.49 (0.31, 0.67) g/dL *P* < 0.001 *I*^2^ = 94%	NR
Dewey et al. (40)	Home fortification	-	1,263 children between 6 months and 2 years of age	Home fortification as treatment verses iron drops	−0.91 (−11.96, 10.14) g/L	NR
De-Regil et al. (38)	Home fortification	2	278 children 6–23 months	Home fortification with MMP verses iron supplements	MD (IV, RE, 95%CI) = −2.36 (−10.30 to 5.59) g/L *I*^2^ = 78%	Low
** <5 YEARS**						
Thompson et al. (26) (end point)	Iron supplementation	9	1,690 children between 2 and 5 years of age	Daily iron supplementation ≥5 days/week verses control	MD (95% CI) = 6.97 (4.21, 9.72) g/L *P* < 0.00001 *I*^2^ = 82%	High
De-Regil et al. (28)	Iron supplementation	9	1,254 apparently healthy children <5 years of age	Intermittent iron supplements verses placebo	MD (IV, RE, 95%CI) = 6.45 (2.36, 10.55) g/L	NR
Eichler et al. (36)	Iron + MMN fortification	8	1,803 Infants and children from 6 months to 5 years of age	Iron + MMN fortification verses non-fortified food	MD (95% CI) = 0.87 (0.57–1.16) g/dL *P* = 0.00 *I*^2^ = 81.5%	NR
Eichler et al. (36)	Iron fortification	13	2,274 Infants and children from 6 months to 5 years of age	Iron fortification of milk and cereals verses non-fortified food	MD (95% CI) = 0.62 (0.34, 0.89) g/dL *P* = 0.00 *I*^2^ = 86.2%	NR
Gera et al. (44) (change in Hb)	Iron supplementation/ fortification	44	Children ≤ 5 years of age	Iron supplementation/ fortification verses control	WMD (RE, 95%CI) = 0.59 (0.43, 0.75)g/dL *P* < 0.001	NR
Cembranel et al. (23)	Iron supplementation	14	Children <5 years of age	Daily doses of FS verses control	MD (95%CI) = 0.56 (0.31, 0.81) mg/dL *P* < 0.001	NR
Kristjansson et al. (41) [change in Hb (g/L)]	Supplementary feeding	5	300 children aged 3 months to 5 years in LMICs	Supplementary feeding verses control	SMD (IV,RE, 95%CI) = 0.49 (0.07–0.91) g/L *I*^2^ = 63%	NR
Matsuyama et al. (30)	Milk fortification	NR	Healthy children aged 6–47 months	Fortified milk verses control	MD (95%CI) = 5·89 (−0·24, 12·02) g/L *P* = 0.06	NR
De-Regil et al. (31) (g/L)	MNP fortification	7	2,023 children between 2 and 5 years of age	Point-of-use fortification of foods with MNP verses no intervention or placebo	MD (IV, RE, 95%CI) = 2.02 (−0.87, 4.92) g/L	NR
Eichler et al. (36)	Iron fortification	5	471 Infants and children from 6 months to 5 years of age	Iron single-fortification verses non-fortified food	MD (95% CI) = 0.20 (−0.05–0.45) g/dL *P* = 0.132 *I*^2^ = 43.4%	NR
Cembranel et al. (23)	Iron supplementation	07	Children <5 years of age	Weekly doses of FS verses control	MD (95%CI) = 0.28 (−0.22, 0.78) mg/dL *P* = 0.273	NR
Cembranel et al. (23)	Iron supplementation	03	Children <5 years of age	Daily doses verses control weekly doses of FS	MD (95%CI) = 0.28 (−0.01, 0.56) mg/dL *P* = 0.057	NR
De-Regil et al. (28)	Iron supplementation	14	2,270 apparently healthy children <5 years of age	Intermittent iron supplements verses daily iron supplements	MD (IV, RE, 95%CI) = −0.75 (−1.80, 0.29) g/L	NR
**CHILDREN** ** <12 YEARS**						
De-Regil et al. (31)	MNP Fortification	11	2,746 preschool and school-age children	Point-of-use fortification of foods with MNP verses no intervention or placebo	MD (95%CI) = 3.37 (0.94, 5.80) g/L	Low
Das et al. (34)	Iron fortification	16	3,832 preschool and school-going children	Iron fortification verses unfortified foods/ regular diet	SMD (95% CI) = 0.46 (0.14, 0.50)	Moderate
Das et al. (34)	MMN fortification	7	1,543 preschool and school-going children	MMN fortification verses unfortified foods/ regular diet	SMD (95% CI) = 0.45 (0.12, 0.79)	Moderate
Mayo-Wilson et al. (22)	Zn supplementation	27	6,024 Children between 6 months and 12 years of age	Zinc supplementation verses no zinc supplementation	SMD (95%CI) = −0.05 (−0.10,0.00) *P* = 0.002 *I*^2^ = 45%	Low
Mayo-Wilson et al. (22)	Zn + Iron supplementation	8	1,341 Children between 6 months and 12 years of age	Zinc with iron supplementation verses zinc alone	SMD (95%CI) = −0.23 (−0.34,−0.12) *P* < 0.0001 *I*^2^ = 79%	Low
De-Regil et al. (28)	Iron supplementation	19	3,032 apparently healthy children <12 years	Intermittent iron supplementation verses placebo/no intervention	MD (IV, RE, 95%CI) = 5.20 (2.51,7.88) g/L *P* = 0.00015 *I*^2^ = 93%	Low
De-Regil et al. (28)	Iron supplementation	7	1,616 apparently healthy children <12 years	Intermittent iron supplementation (0–3 months duration) verses placebo/no intervention	MD (IV, RE, 95%CI) = 5.16 (0.90, 9.36) g/L	NR
De-Regil et al. (28)	Iron supplementation	12	1,416 apparently healthy children <12 years	Intermittent iron supplementation (>3 months duration) verses placebo/no intervention	MD (IV, RE, 95%CI) = 5.13 (2.82, 7.51) g/L	NR
De-Regil et al. (28)	Iron supplementation	11	1,699 apparently healthy children <12 years	Intermittent iron only supplementation verses placebo/no intervention	MD (IV, RE, 95%CI) = 4.41 (1.32, 7.50) g/L	NR
De-Regil et al. (28)	Iron supplementation	4	756 apparently healthy children <12 years	Intermittent iron folic acid supplementation verses placebo/no intervention	MD (IV, RE, 95%CI) = 3.36 (1.51, 5.21) g/L	NR
De-Regil et al. (28)	Iron +Zinc supplementation	1	77 apparently healthy children <12 years	Intermittent iron with zinc supplementation verses placebo/no intervention	MD (IV, RE, 95%CI) = −1.60 (−8.09, 4.89) g/L	NR
De-Regil et al. (28)	Iron +Vit C supplementation	1	50 apparently healthy children <12 years	Intermittent iron with vitamin C supplementation verses placebo/no intervention	MD (IV, RE, 95%CI) = 20.70 (17.51, 23.89) g/L	NR
De-Regil et al. (28)	Iron +MMN supplementation	4	450 apparently healthy children <12 years	Intermittent iron with MMN supplementation verses placebo/no intervention	MD (IV, RE, 95%CI) = 5.47 (0.32, 10.61) g/L	NR
De-Regil et al. (28)	Iron ± MMN supplementation	19	2,851 apparently healthy children <12 years	Intermittent iron supplements ± MMN verses daily iron supplements ± MMN	MD (IV, RE, 95%CI) = −0.60(−1.54,0.35) g/L *P* = 0.22 *I*^2^ = 56%	Low
De-Regil et al. (28)	Iron supplementation	11	1,455 apparently healthy children <12 years	Intermittent iron supplements verses daily iron supplements (0–3 months duration)	MD (IV, RE, 95%CI) = 0.47 (−0.91, 1.84) g/L	NR
De-Regil et al. (28)	Iron supplementation	8	1,387 apparently healthy children <12 years	Intermittent iron supplements verses daily iron supplements (> 3 months duration)	MD (IV, RE, 95%CI) = −1.14 (−2.07,−0.22) g/L	NR
De-Regil et al. (28)	Iron supplementation	15	2,144 apparently healthy children <12 years	Intermittent iron supplements verses daily iron supplements (Iron alone)	MD (IV, RE, 95%CI) = −0.51 (−1.61, 0.59) g/L	NR
De-Regil et al. (28)	Iron supplementation	2	408 apparently healthy children <12 years	Intermittent iron supplements verses daily iron supplements (With folic acid)	MD (IV, RE, 95%CI) = −2.26 (−4.30,−0.22) g/L	NR
De-Regil et al. (28)	Iron supplementation	3	299 apparently healthy children <12 years of age	Intermittent iron supplements verses daily iron supplements (With MMN)	MD (IV, RE, 95%CI) = 0.61 (−2.04, 3.26) g/L	NR
De-Regil et al. (28)	Iron supplementation	10	1,778 apparently healthy children between 5 and12 years of age	Intermittent iron supplements verses placebo or no intervention	MD (IV, RE, 95%CI) = 4.04 (0.30, 7.78) g/L	NR
De-Regil et al. (28)	Iron supplementation	5	581 apparently healthy children between 5 and 12 years of age	Intermittent iron supplements verses daily iron supplements	MD (IV, RE, 95%CI) = −0.31 (−2.59, 1.97) g/L	NR
**CHILDREN** ** <18 YEARS OF AGE**						
Girum et al. (47) (change in Hb)	Deworming	8	10,05,239 school children from Asia and Africa	Deworming verses control	WMD (FE, 95%CI) = 1.62 (1.01–2.25) g/dL *P* = 0.873 *I*^2^ = 0%	NR
Low et al. (24)	Iron supplementation	28	6,545 primary-school–aged children between 3.3 and 15 years	Iron supplementation verses placebo/anti-helminthic therapy/Zinc/multi-vitamin	MD (95%CI) = 8.38 (6.21–10.56) g/L *P* < 0.001 *I*^2^ = 97%	NR
Low et al. (20) (end of treatment)	Iron supplementation	10	3,220 participants between 12 and 18years	Daily oral iron supplementation with or without other vitamins (folic acid or vitamin C)/verses control/placebo	MD (IV, RE, 95%CI) = 6.99 (3.85, 10.13) g/L *P* = 0.00001 *I*^2^ = 95%	NR for subgroup
Aaron et al. (32)	MMN fortification	8	3,835 apparently healthy children between 5 and 18 years of age	MMN fortified beverages verses non-fortified beverages	2.79 (1.19, 4.33) g/L *P* = 0.004 *I*^2^ = 92%	Moderate
Ramakrishnan et al. (29)	Iron interventions	16	2,542 children between 6 months and 18 years	Iron interventions verses control	WMD (95%CI) = 1.49 (0.46, 2.51)	NR
Gera et al. (44) (change in Hb)	Iron supplementation/fortification	91	12,198 children <18 years of age	Iron supplementation/ fortification verses control	WMD (95%CI) = 0.74 (0.61–0.87) g/dL *P* < 0.001	NR
Gera et al. (44) (change in Hb)	Iron supplementation	82	Children <18 years of age	Iron supplementation verses control	WMD (95%CI) = 0.79 (0.65, 0.94) g/dL *P* < 0.001	NR
Gera et al. (44) (change in Hb)	Iron fortification	9	Children <18 years of age	Iron fortification verses control	WMD (RE, 95%CI) = 0.25 (0.02, 0.52) g/dL *P* = 0.065	NR
Gera et al. (44) (change in Hb)	Iron supplementation/ fortification	47	Children >5 years of age	Iron supplementation/ fortification verses control	WMD (RE, 95%CI) = 0.88 (0.67, 1.08) g/dL *P* < 0.001	NR
Gera et al. (44) (change in Hb)	Iron supplementation/fortification	13	Children <18 years of age from developed countries	Iron supplementation/ fortification verses control	WMD (RE, 95%CI) = 0.46 (0.13, 0.78) g/dL *P* = 0.007	NR
Gera et al. (44) (change in Hb)	Iron supplementation/ fortification	78	Children <18 years of age from developing countries	Iron supplementation/ fortification verses control	WMD (RE, 95%CI) = 0.78 (0.64, 0.93) g/dL *P* < 0.001	NR
Das et al. (34)	Vitamin A fortification	2	1,538 children <18 years of age	Vitamin A fortification verses unfortified foods/ regular diet	SMD (95% CI) = 0.48 (0.07, 0.89)	Low
Das et al. (34)	Iron fortification	20	4,176 children from UMHICs	Iron fortification verses unfortified foods/ regular diet	SMD (95% CI) = 0.67 (0.36, 0.97)	Moderate
Das et al. (34)	MMN fortification	8	1,769 children from LMICs	MMN fortification verses unfortified foods/ regular diet	SMD (95% CI) = 0.50 (0.21, 0.78)	Moderate
Das et al. (34)	MMN fortification	6	1,282 children from UMHICs	MMN fortification verses unfortified foods/ regular diet	SMD (95% CI) = 1.25 (0.45, 2.06)	Moderate
Das et al. (34)	Zinc fortification	NR	Children below 18 years	Zinc fortification verses unfortified foods/ regular diet	SMD (95% CI) = −0.11 (−0.52, 0.31)	Low
Gera et al. (43) (change in Hb)	Iron and MMN supplementation	25	4,981 children <15 years from developing countries	Iron and MMN supplementation verses placebo	WMD (RE, 95 % CI) = 0.65 (0.50, 0.80) g/L *P* < 0.001 *I*^2^ = 89.6 %	NR
Gera et al. (43) (change in Hb)	Iron and MMN supplementation	13	1,483 children <15 years from developing countries	Iron and MMN supplementation verses iron supplementation alone	WMD (95 % CI) = 0.14 (0.00, 0.28) g/L *P* = 0.044 *I*^2^ = 76.0%	NR
Gera et al. (43)	Iron and micronutrient supplementation	18	Children <15 years from developing countries	Iron and micronutrient supplementation verses placebo	WMD (RE, 95%CI) = 0.69 (0.48, 0.91) g/L *P* < 0.001	NR
Gera et al. (43)	Iron and micronutrient fortification	15	Children <15 years from developing countries	Iron and micronutrient fortification verses placebo	WMD (RE, 95%CI) = 0.61 (0.40, 0.81) g/L *P* < 0.001	NR
Gera et al. (43)	Iron and micronutrient supplementation	9	Children <15 years from developing countries	Iron and micronutrient supplementation verses iron	WMD (95 % CI) = 0.27 (0.13, 0.41) g/L *P* < 0.001 *I*^2^ = 8.5%	NR
Gera et al. (43)	Iron and micronutrient fortification	6	Children <15 years from developing countries	Iron and micronutrient fortification verses iron	WMD (95 % CI) = 0.06 (20.15, 0.27) g/L *P* = 0.55 *I*^2^ = 89.3%	NR
De-Regil et al. (31)	MNP Fortification	3	524 children aged ≥5 years	Point-of-use fortification of foods with MNP verses no intervention or placebo	MD (IV, RE, 95%CI) = 7.86 (−0.76, 16.49) g/L	NR
Neuberger et al. (21) (end of treatment)	Iron supplementation	16	5,261Children <18 years of age from malaria endemic areas	Iron vs. placebo or no treatment	MD (IV, RE, 95%CI) = 0.75 (0.48, 1.01) g/dL *P* < 0.00001 *I*^2^ = 93%	NR
Neuberger et al. (21) (change in Hb)	Iron supplementation	12	2,462 children <18 years of age from malaria endemic areas	Iron vs. placebo or no treatment	MD (IV, RE, 95%CI) = 0.67 (0.42, 0.92) g/dL *P* < 0.00001 *I*^2^ = 82%	NR
Neuberger et al. (21) (end of treatment)	Iron plus folic acid supplementation	1	124 children <18 years of age from malaria endemic areas	Iron plus folic acid vs. placebo	MD (IV, RE, 95%CI) = 0.90 (0.51, 1.29) g/dL *P* < 0.00001 *I*^2^ = NA	NR
Neuberger et al. (21) (end of treatment)	Iron plus anti-malarial treatment	1	151 children <18 years of age from malaria endemic areas	Iron plus anti-malarial treatment vs. placebo	MD (IV, RE, 95%CI) = 0.91 (0.47, 1.35) g/dL *I*^2^ = NA	NR
Athuman et al. (50) (mean change in Hb)	Anti-malarial treatment	4	1,672 children with anemia in Malaria-endemic areas	Intermittent preventive antimalarial treatment verses placebo (IPT ± iron and folic acid verses placebo ± iron and folic acid)	MD (IV, FE) = 0.32 (0.19, 0.45) g/dL *I*^2^ = 18%	Low
Athuman et al. (50) (mean Hb)	Anti-malarial treatment	4	1,672 children with anemia in Malaria-endemic areas	Intermittent preventive antimalarial treatment verses placebo (IPT ± iron and folic acid verses placebo ± iron and folic acid)	MD (IV, FE) = 0.35 (0.06, 0.64) g/dL *I*^2^ = 76%	Low
Taylor-Robinson et al. (46)	Deworming	4	1,992 children <16 years in areas endemic for intestinal helminthes	Deworming drugs verses no intervention	MD (IV,FE, 95%CI) = 0.02 (−0.05, 0.09) g/dL *P* > 0.05	Low
Taylor-Robinson et al. (46)	Deworming	2	108 children <16 years in areas endemic for intestinal helminthes or children screened for infection	Single dose deworming drugs verses no intervention	MD (IV,FE, 95%CI) = 0.37 (0.10, 0.64) g/dL *P* = 0.008 *I*^2^ = 0%	Low
Taylor-Robinson et al. (46)	Deworming	2	658 children <16 years in areas endemic for intestinal helminthes (moderate prevalence)	Single dose deworming drugs (targeted intervention) verses no intervention	MD (IV,FE, 95%CI) = 0.06 (−0.06, 0.17) g/dL *P* = 0.34 *I*^2^ = 0%	NR
Taylor-Robinson et al. (46)	Deworming	2	1,334 children <16 years in areas endemic for intestinal helminthes (low prevalence)	Single dose deworming drugs (targeted intervention) verses no intervention	MD (IV,FE, 95%CI) = 0.00 (−0.08, 0.08) g/dL *P* = 0.92 *I*^2^ = 0%	NR
Taylor-Robinson et al. (46) (in first year)	Deworming	4	807 children <16 years in areas endemic for intestinal helminthes	Multiple dose deworming drugs verses no intervention	MD (IV,FE, 95%CI) = 0.01 (0.13, 0.14) g/dL	Low
Taylor-Robinson et al. (46) (in first year)	Deworming	2	464 children <16 years in areas endemic for intestinal helminthes (moderate prevalence)	Multiple dose deworming drugs (targeted intervention) verses no intervention	MD (IV,FE, 95%CI) = 0.02 (−0.15, 0.19) g/dL *P* = 0.82 *I*^2^ = 0%	NR
Taylor-Robinson et al. (46) (in first year)	Deworming	2	343 children <16 years in areas endemic for intestinal helminthes (low prevalence)	Multiple dose deworming drugs (targeted intervention) verses no intervention	MD (IV,FE, 95%CI) = −0.06 (−0.28, 0.17) g/dL *P* = 0.62 *I*^2^ = 0%	NR
Taylor-Robinson et al. (46) (after first year)	Deworming	2	1,365 children <16 years in areas endemic for intestinal helminthes (low prevalence)	Multiple dose deworming drugs (targeted intervention) verses no intervention	MD (IV,FE, 95%CI) = −0.02 (−0.30, 0.27) g/dL *P* = 0.91 *I*^2^ = 0%	Very low
Huang et al. (48) (changes in Hb)	*H. pylori* treatment	4	<18 years of age	*H. pylori* treatment for eradication verses placebo	WMD (IV, RE, 95%CI) = 11.77 (2.40, 21.15) g/L *P* = 0.01 *I*^2^ = 90%	NR

In infants, MMN fortification showed maximum improvement in Hb (SMD = 1.05, 95%CI = 0.48–1.63; seven studies; 1,508 participants; *moderate QoE*) (34), followed by iron fortification (SMD = 0·81, 95%CI = 0.31–1.31; 12 studies; 1,834 infants; *moderate QoE*) (34).

In children under 2 years old, daily iron supplementation showed significant improvements in Hb (MD = 7.22 g/L, 95%CI = 4.87–9.57; *I*^2^ = 94%; 26 studies; 5,479 participants 4M−2Y) (25), followed by MMN fortification (MD = 5.87 g/L, 95%CI = 3.25–8.49, six studies, 1,447 participants 6M−2Y) (38), and home fortification (5.06 g/L; 95%CI = 2.29–7.83, 22 studies, 2,449 participants 6M−2Y) (40). Home fortification did not show a significant impact on Hb levels as compared with iron drops (−0.91 g/L; 95%CI = −11.96–10.14; 1,263 participants 6M−2Y) (40) or iron supplements (MD = −2.36 g/L; 95%CI = −10.30–5.598, *I*^2^ = 78%, two studies, 278 participants 6M−2Y) (38).

In children under 5 years old, maximum improvement in Hb levels was seen with MMN fortification (MD = 0.87 g/dL, 95%CI = 0.57–1.16; *I*^2^ = 82%; eight studies; 1,803 participants 6M−5Y) (36), followed by daily iron supplementation (MD = 6.97 g/L, 95%CI = 4.21–9.72; *I*^2^ = 82%; nine studies; 1,690 participants 2–5Y; *high QoE*) (26), intermittent iron supplementation (MD = 6.45 g/L, 95%CI = 2.36–10.55; nine studies, 1,254 apparently healthy participants <5Y) (28), iron fortification of milk and cereals (MD = 0.62 g/dL, 95%CI = 0.34–0.89; *I*^2^ = 86.2%; 13 studies, 2,274 participants 6M−5Y) (36), and daily FS supplementation (MD = 0.56 mg/dL, 95%CI = 0.31–0.81; 14 studies; participants <5Y) (23). Insignificant changes were observed with fortified milk (MD = 5.89 g/L, 95%CI = −0.24–12.02) (30), point-of-use fortification of food with MNP (MD = 2.02 g/L, 95%CI = −0.87 to 4.92) (31), single iron fortification (MD = 0.20 g/dL, 95%CI = −0.05 to 0.45) (36), and weekly FS supplementation (MD = 0.28 mg/dL; 95%CI = −0.22 to 0.78) (23).

The maximum effect on Hb in preschool and school-age children was seen with iron fortification (SMD = 0.46, 95%CI = 0.24–0.67; 16 studies; 3,832 participants; *moderate QoE*) (34) and MMN fortification (SMD = 0.45, 95%CI = 0.12–0.79; seven studies; 1,543 participants; *moderate QoE*) (34) followed by point-of-use fortification with MMN (MD = 3.37 g/L, 95%CI = 0.94–5.80; 11 studies; 2,746 participants; *low QoE*) (31). In apparently healthy children under 12 years of age, intermittent supplementation with iron alone or in combination with other nutrients effectively increased Hb concentrations, and this positive response did not differ between frequency or duration of supplementation or age of children (28).

In children under 15 years of age, daily iron supplementation (MD = 8.38 g/L, 95%CI = 6.21–10.56; *I*^2^ = 97%; 28 studies; 6,545 participants 3.3–15Y) (24) and iron with micronutrient supplementation (WMD = 0.69 g/L, 95%CI = 0.48–0.91; 18 studies; participants <15Y) showed better improvements in Hb than iron with micronutrient fortification (WMD = 0.61 g/L, 95%CI = 0.40–0.81; 15 studies; participants <15Y) (43). A school-based deworming program resulted in a decreased prevalence of anemia (WMD = 1.62, 95%CI = 1.01–2.25; *I*^2^ = 0%; eight studies; 1,005,239 participants) (47).

In children under 18 years of age, point-of-use fortification of food with MMP showed maximum though insignificant improvements in Hb levels (MD = 7.86 g/L, 95%CI = −0.76–16.49; three studies; 524 participants ≥5Y) (31). Daily oral iron supplementation with or without other vitamins (MD = 6.99 g/L, 95%CI = 3.85–10.13; *I*^2^ = 95%; 10 studies; 3,220 participants 12–18Y) (20) and MMN-fortified beverages (2.76 g/L, 95%CI = 1.19–4.33; eight studies; 3,855 apparently healthy participants 5–18Y) showed significant improvements (32). Also, there was a substantially higher increase in Hb concentration from iron interventions (WMD = 1.49, 95%CI = 0.46–2.51; 16 studies; 2,542 participants 6M−18Y) (29) than oral iron supplementation (WMD = 0.79 g/dL, 95%CI = 0.65–0.94; 82 studies) and iron fortification (WMD = 0.25 g/dL, 95%CI = 0.02–0.52; nine studies; participants <18Y) (44). Fortification of food with vitamin A substantially increased Hb concentrations in children under 18 years of age (SMD = 0.48, 95%CI = 0.07 = 0.89; two studies; 1,538 participants <18Y), with iron (SMD = 0.67, 95%CI = 0.36–0.97; 20 studies; 4,176 participants <18Y from UMHICs), and with MMN (SMD = 0.50, 95%CI = 0.21–0.78; eight studies; 1,769 participants <18Y from LMICs) (34).

In children under 18 years of age residing in malaria-endemic areas, iron with antimalarial treatment (MD = 0.91 g/dL, 95%CI = 0.47–1.35; one study; 151 participants <18Y) and iron with FA supplementation (MD = 0.90 g/dL, 95%CI = 0.51–1.29; one study; 124 participants <18Y) showed better results as compared with iron-only supplementation (MD = 0.75 g/dL, 95%CI = 0.48–1.01; *I*^2^ = 93%; 16 studies; 5,261 participants <18Y) (21). There is low-quality evidence that community deworming has little or no effect (MD = 0.02 g/dL, 95%CI = −0.05 to 0.09; *I*^2^ = 0%; two studies; 464 participants <16Y) and that, in programs that treat only infected children, a single dose of deworming may improve Hb (MD = 0.37 g/dL, 95%CI = 0.10–0.64; two trials; 108 participants <16Y; *low QoE*) (46). Additional *H. pylori* eradication therapy along with iron administration provided better improvements in iron deficiency anemia than iron administration alone (WMD = 11.77 g/L, 95%CI = 2.40–21.15; *I*^2^ = 90%; four studies; participants <18Y) (48).

#### Adverse Events

A total of six reviews addressed the adverse effects of different interventions for preventing, treating, and controlling anemia (21, 22, 24, 25, 28, 31) (Table 4). We did not find any review that assessed the adverse effects of antimalarial intervention, deworming, *H. pylori* treatment, or WASH interventions.

**Table 4:** Findings for adverse events of different interventions for addressing childhood anemias in included systematic reviews.

**SR**		**#Included studies**	**#Participants**	**Comparison**	**Meta-analysis**	**Quality of evidence**
**CHILDREN** ** <2 YEARS**						
Pasricha et al. (25) (vomiting)	Iron supplements	3	1,020 children between 4 and 23 months	Iron supplementation verses control	RR (95%CI) = 1.38 (1.10–1.73) *P* = 0.006 *I*^2^ = 1%	NR
Pasricha et al. (25) (diarrhea prevalence)	Iron supplements	6	1,697 children between 4 and 23 months	Iron supplementation verses control	RR (95%CI) = 1.03 (0.86–1.23) *P* = 0.78 *I*^2^ = 0%	NR
Pasricha et al. (25) (constipation)	Iron supplements	2	570 children between 4 and 23 months	Iron supplementation verses control	RR (95%CI) = 0.54 (0.05–5.83) *P* = 0.49 *I*^2^ = 77%	NR
Pasricha et al. (25) (any adverse events)	Iron supplements	3	912 children between 4 and 23 months	Iron supplementation verses control	RR (95%CI) = 1.10 (0.98–1.25) *P* = 0.12 *I*^2^ = 0%	NR
**CHILDREN** ** <12 YEARS**						
De-Regil et al. (28)	Iron supplements	1	53 apparently healthy children <5 years of age	Intermittent supplementation verses placebo or no intervention	RR (M-H, RE, 95%CI) = 3.87 (0.19, 76.92)	NR
De-Regil et al. (28)	Iron supplements	4	895 apparently healthy children <5 years of age	Intermittent iron supplements verses daily iron supplements	RR (M-H, RE, 95%CI) = 0.60 (0.19, 1.87)	NR
De-Regil et al. (28)	Iron supplements	1	53 apparently healthy children between 5 and 12 years of age	Intermittent supplementation verses placebo or no intervention	RR (M-H, RE, 95%CI) = 3.87 (0.19, 76.92)	NR
De-Regil et al. (28) (any AE)	Iron supplements	1	53 apparently healthy children <12 years	Intermittent supplementation with iron alone or with other nutrients verses placebo or no intervention	RR (M-H, FE, 95%CI)= 3.87 (0.19, 76.92) *P* = 0.37 *I*^2^ =NA	NR
De-Regil et al. (28) (nausea)	Iron supplements	1	64 apparently healthy children <12 years	Intermittent supplementation with iron alone or with other nutrients verses placebo or no intervention	RR (M-H, RE, 95%CI) 2.82 (0.12, 66.82) *P* = 0.52 *I*^2^ =NA	NR
De-Regil et al. (28) (any AE)	Iron supplements	4	895 apparently healthy children <12 years	Intermittent iron supplements ± other micronutrients verses daily iron supplements ± other micronutrients	RR (M-H, RE, 95%CI)= 0.60 (0.19, 1.87) *P* = 0.38 *I*^2^ = 87%	NR
De-Regil et al. (28) (diarrhea)	Iron supplements	1	122 apparently healthy children <12 years	Intermittent iron supplements ± other micronutrients verses daily iron supplements ± other micronutrients	RR (M-H, RE, 95%CI)= 1.17 (0.60, 2.28) *P* = 0.65 *I*^2^ =NA	NR
Mayo-Wilson et al. (22) (incidence of vomiting)	Zn/Iron supplementation	5	4,095 Children between 6 months and 12 years of age	Zinc supplementation verses no zinc supplementation	RR (95%CI) = 1.68 (1.61, 1.75) *P* < 0.00001 *I*^2^ = 85%	Low
Mayo-Wilson et al. (22) (prevalence of vomiting)	Zn/Iron supplementation	4	35,192 Children between 6 months and 12 years of age	Zinc supplementation verses no zinc supplementation	RR (95%CI) = 1.29 (1.14, 1.46) *P* = 0.18 *I*^2^ = 37%	Low
Mayo-Wilson et al. (22) (study withdrawal)	Zn/Iron supplementation	6	4,263 Children between 6 months and 12 years of age	Zinc supplementation verses no zinc supplementation	RR (95%CI) = 1.75 (0.93, 3.32) *P* = 0.28 *I*^2^ = 21%	Low
Mayo-Wilson et al. (22) (study withdrawal)	Zn/Iron supplementation	2	557 Children between 6 months and 12 years of age	Zinc with iron supplementation verses zinc alone supplementation	RR (95%CI) = 1.41 (0.91, 2.18) *P* = 0.78 *I*^2^ = 0%	Low
De-Regil et al. (31) (adverse effects)	Food fortification	1	90 Preschool and school-age children	Point-of-use fortification of foods with MNP verses no intervention or placebo	RR (M-H, RE, 95%CI) = 1.09 (0.16–7.42) *P* > 0.05 *I*^2^ =NA	Moderate
De-Regil et al. (31) (diarrhea)	Food fortification	2	366 Preschool and school-age children	Point-of-use fortification of foods with MNP verses no intervention or placebo	RR (M-H, RE, 95%CI) = 0.97 (0.53, 1.78) *P* = 0.93 *I*^2^ = 0%	Low
Low et al. (24) (gastrointestinal upset)	Iron supplementation	4	576 primary-school–aged children between 3.3 and 15 years	Iron supplementation verses placebo/antihelminthic therapy/Zinc/multi-vitamin	RR (95%CI) = 1.30 (0.89–1.91) *P* = 0.2 *I*^2^ = 0%	NR
Low et al. (24) (constipation)	Iron supplementation	2	202 primary-school–aged children between 3.3 and 15 years	Iron supplementation verses placebo/antihelminthic therapy/Zinc/multi-vitamin	RR (95%CI) = 3.44 (0.66–19.68) *P* = 0.2 *I*^2^ = 6%	NR
Low et al. (24) (vomiting)	Iron supplementation	2	202 primary-school–aged children between 3.3 and 15 years	Iron supplementation verses placebo/antihelminthic therapy/Zinc/multi-vitamin	RR (95%CI) = 0.86 (0.13–5.67) *P* = 0.9 *I*^2^ = 0%	NR
Neuberger et al. (21) (diarrheal episodes per patient-month by Zn supplements)	Iron supplementation	8	23,912 children in malaria endemic areas	Iron vs. placebo or no treatment	RR (IV, FE, 95%CI) = 1.15 (1.06, 1.26) *P* = 0.0014 *I*^2^ = 40%	NR
Dewey et al. (40) (diarrhea)	Food Fortification	8	808 children between 5.5 and 18 years	Treatment home fortification verses control	−0.34 (−0.78, 0.03) *P* = 0.02	NR
Dewey et al. (40) (diarrhea)	Food Fortification	10	1,195 children between 5.5 and 18 years	Preventive home fortification verses control	RR (95%CI) = 1.07 (0.78, 1.47) *P* = 0.72	NR
Neuberger et al. (21) (diarrheal episodes per patient-month without Zn)	Iron supplementation	7	17,566 children in malaria endemic areas	Iron vs. placebo or no treatment	RR (FE, 95%CI) = 0.99 (0.87, 1.13) *P* = 0.93 *I*^2^ = 0%	NR
Neuberger et al. (21) (diarrheal episodes per patient-month with Zn)	Iron supplementation	3	6,346 children in malaria endemic areas	Iron vs. placebo or no treatment	RR (IV, FE, 95%CI) = 1.29 (1.15, 1.44) *P* = 0.000017 *I*^2^ = 30%	NR

In children between 4 months and 2 years, there is an uncertain effect of risk of adverse events with iron supplements as compared with controls with a 10% increase in the risk of any adverse event (RR = 1.10, 95%CI = 0.98–1.25; *I*^2^ = 0%; three studies, 912 participants), 38% increase in the risk of vomiting (RR = 1.38, 95%CI = 1.10–1.73; *I*^2^ = 1%; three studies, 1,020 participants), and 3% increase in the risk of diarrhea (RR = 1.03, 95%CI = 0.86–1.23; *I*^2^ = 0%; six studies; 1,697 participants) (25).

In apparently healthy children under 12 years of age, intermittent supplementation of iron alone or with other nutrients showed a 3.87-times risk of any adverse events (RR = 3.87, 95%CI = 0.19–76.92; one study; 53 participants) (28). However, the risk of any adverse events was 40% less (RR = 0.60, 95%CI = 0.19–1.87; *I*^2^ = 87%; four studies; 895 participants), and the risk of diarrhea was 1.17 times higher (RR = 1.17, 95%CI = 0.60–2.28; *I*^2^ = 0%; one study; 122 participants) with intermittent iron supplements with or without other micronutrients as compared with daily iron supplements with or without other micronutrients (28). There is an insignificant effect of food fortification on adverse effects (RR = 1.09, 95%CI = 0.16–7.42; one study; 90 participants; *moderate QoE*) and diarrhea (low QoE) in preschool and school-age children (31). The risk of gastrointestinal upset was 1.3 times higher (RR = 1.30, 95%CI = 0.89–1.91; *I*^2^ = 0%; four studies; 576 participants), constipation was 3.44 times higher (RR = 3.44, 95%CI = 0.66–19.68; *I*^2^ = 6%; two studies, 202 participants), and vomiting was 14% lower (RR = 0.86, 95%CI = 0.13–5.67; *I*^2^ = 0%; two studies; 202 participants) with iron supplements in school-aged children between 3.3 and 15 years (24).

In children under 18 years old residing in malaria endemic areas, there is a significantly higher risk of diarrheal episodes per patient-month by 15% with iron supplements (RR = 1.15, 95%CI = 1.06–1.26; eight studies; 23,912 participants) and by 29% with Zn supplements (RR = 1.29, 95%CI = 1.15–1.44; *I*^2^ = 30%; three studies, 6,346 participants) (21).

## Discussion

We evaluated 31 SRs that assessed the effects of different interventions for addressing anemia in children and adolescents. The highest number of SRs addressed food fortification and iron supplementation. None of the reviews were from India or included participants from India. Overall, the quality of the evidence reported by the SRs varied between moderate to low (Supplementary File 2).

In infants, iron fortification showed better results as compared with MMN fortification (30, 34). In children between 4 months and 2 years of age; home fortification, when used as a preventive measure, showed significant reduction in the risk of anemia followed by iron supplementation (25, 38, 40). However, when used as a treatment measure, home fortification insignificantly increased the risk of anemia (40). This difference in the effect of home fortification when used as a preventive measure and as a treatment measure might be because the prevention trials were conducted on unselected populations, and the participants in the control group did not receive any treatment. On the contrary, the therapeutic trials were conducted on anemic subjects, and the participants in the control received medicinal iron drops (40).

In children under 5 years old and in school-age children, fortification of food with iron and MMN showed better improvement in Hb (*moderate QoE*) (34, 36). In apparently healthy children under 12 years old, intermittent supplementation of iron alone or in combination with other nutrients significantly increased Hb concentrations, and this positive response did not differ with frequency or duration of supplementation or age of children (28). The risk of anemia is higher with intermittent iron/FA/MMN supplementation as compared with daily iron/FA/MMN supplementation (28). A school-based deworming program resulted in a decrease in the prevalence of anemia in children (47). Programs that combined interventions, such as deworming with iron and nutrition supplementation, led to better effects as compared with single interventions (47). Daily oral iron supplementation with or without other vitamins (20, 24) and MMN-fortified beverages (32) showed maximum reduction in the risk of anemia in children between 3.5 and 18 years. In malaria endemic areas, iron supplementation with antimalarial treatment reduced the risk of anemia (21). There is low-quality evidence that community deworming has little or no effect (46).

In summary, results were favorable for iron with MMN fortification until school age and iron with MMN supplementation in older children with a clear reduction in the risk of anemia and increase in Hb among the intervention groups of individual SRs. However, other interventions reported by SRs were inconclusive and all SRs suggested further research on the same.

The chances of developing anemia were less in children who received daily supplements as compared with those who received intermittent supplements. However, studies suggest that, in places and settings in which daily supplementation is not practical, intermittent supplementation can be used as a public health intervention to address childhood anemia. We did not find any review that assessed the effect of treatment of deworming, *H. pylori* infection, or WASH intervention on anemia status.

Limitations of the review: This overview examined the available evidence with respect to interventions addressing childhood and adolescent anemia without any restrictions on the type of interventions or language of publication. We did not restrict the search to Cochrane reviews, and inclusion of non-Cochrane reviews makes it more comprehensive. However, wide physiological variations during different stages of childhood could make the uniform application of interventions difficult. We acknowledge that not all SRs included in this overview were up to date. We found limited reviews on deworming, antimalarial interventions, *H. pylori* intervention, and WASH intervention, and hence, there is a need for primary studies in these areas. Some SRs did not consider socio-economic or nutritional status at baseline or did not report this clearly, which could have had an influence on some findings. In some reviews, there were too few data to reach a firm conclusion.

Strengths of the review: We estimate the potential bias in this overview as low. We followed the Cochrane handbook for methodology (19). The search was as comprehensive as possible. Screening of studies, data extraction, and assessing the methodological quality of reviews were done in duplicate. The authors of this review are from diverse disciplines (e.g., public health, biostatistics, nutrition, physiology, and maternal and child health), and this internal diversity may be an asset of this overview.

After an extensive literature search, we did not come across any overview of SRs that has addressed this area. This overview can help healthcare providers, consumers, public health specialists, and decision makers by providing a “bird's-eye view” of the efficacy of different public health interventions across reviews and age groups.

### Implications of All the Available Evidence

This overview identified interventions that had favorable effect sizes (which public health experts might contemplate using) as well as interventions that were mostly ineffective (which public health experts might want to restrict). More research is needed to assess the effect of deworming, antimalarial intervention, *H. pylori* treatment, and WASH intervention on anemia. Also, better recording of adverse events in primary studies is needed.

## Conclusion

Results were favorable for iron and MMN fortification and supplementation with a clear reduction in the risk of anemia and increase in Hb levels across all age groups. Other interventions reported by the SRs were inconclusive and suggest further research. MMN improves outcomes better than single micronutrients. Also, better recording of adverse effects of different intervention is needed.

## Data Availability Statement

The original contributions presented in the study are included in the article/Supplementary Material, further inquiries can be directed to the corresponding author/s.

## Author Contributions

AS envisioned the idea of the review. RV developed and ran the search strategies. AS and PM developed the protocol. PM and RH screened title and abstract. RH and NK screened full texts. BU resolved disagreement among primary reviewers (for screening) through discussion. AS, PM, and MK extracted data. AS and PM assessed the methodological quality of included SRs. NN, AG, and SQ provided technical advice. MK and PM drafted the manuscript with input from AS, BU, NN, AG, and SQ. All authors have contributed significantly to this overview, read the manuscript, and participated in writing and revision.

## Conflict of Interest

The authors declare that the research was conducted in the absence of any commercial or financial relationships that could be construed as a potential conflict of interest.
