# Supplementary material for: Interventions for Addressing Anemia Among Children and Adolescents: An Overview of Systematic Reviews
Source: Front Pediatr. 2021 Feb 16;8:549549. doi: 10.3389/fped.2020.549549 (PMC7921152; doi:10.3389/fped.2020.549549)
Supplement: Supplementary file 1 [file Data_Sheet_1.docx]

# Supplementary File 1: CENTRAL Search strategy

[Cochrane Database of Systematic Reviews](https://www.cochranelibrary.com/): Issue 2 of 12, February 2019 = 2428 SRs

Last Saved: 05/02/2019 14:14:59

ID Search

#1 child*

#2 children

#3 infant*

#4 toddler*

#5 adolescen*

#6 teenager*

#7 youngster*

#8 pre-school

#9 "school-going"

#10 girl*

#11 boy*

#12 MeSH descriptor: [Child] explode all trees

#13 MeSH descriptor: [Infant] explode all trees

#14 MeSH descriptor: [Adolescent] explode all trees

#15 #1 OR #2 OR #3 OR #4 OR #5 OR #6 OR #7 OR #8 OR #9 OR #10 OR #11 OR #12 OR #13 OR #14

#16 supplement*

#17 fortification

#18 fortified

#19 diversification

#20 “nutritional intervention*”

#21 micronutrient*

#22 anti-helminth

#23 "helminth control"

#24 deworming

#25 WASH

#26 WIFS

#27 MeSH descriptor: [Dietary Supplements] explode all trees

#28 MeSH descriptor: [Food, Fortified] explode all trees

#29 MeSH descriptor: [Hand Disinfection] explode all trees

#30 #16 OR #17 OR #18 OR #19 OR #20 OR #21 OR #22 OR #23 OR #24 OR #25 OR #26 OR #27 OR #28 OR #29

#31 #15 AND #30
