# Supplementary material for: Interventions for Addressing Anemia Among Children and Adolescents: An Overview of Systematic Reviews
Source: Front Pediatr. 2021 Feb 16;8:549549. doi: 10.3389/fped.2020.549549 (PMC7921152; doi:10.3389/fped.2020.549549)
Supplement: Supplementary file 2 [file Data_Sheet_2.docx]

**Supplementary File 2**

**S2 Table: Quality assessment of included systematic reviews by R-AMSTAR tool**

| Study (SR) | Was an  ’a priori’  design provided? | Was there duplicate  study selection  and data extraction? | Was a comprehensive literature search performed? | Was the status of publication (i.e. grey literature) used as an inclusion criterion? | Was a list of studies (included and excluded) provided? | Were the characteristics of the included studies provided? | Was the scientific quality of the included studies assessed and documented? | Was the scientific quality of the included studies used appropriately in formulating conclusions? | Were the methods used to combine the findings of studies appropriate? | Was the likelihood of publication bias (a.k.a. “file drawer” effect) assessed? | Was the conflict of interest stated? | Total score |
| --- | --- | --- | --- | --- | --- | --- | --- | --- | --- | --- | --- | --- |
| Low et al.,[20] | 4 | 4 | 4 | 4 | 4 | 4 | 4 | 4 | 4 | 2 | 2 | 40 |
| Neuberger et al.,[21] | 4 | 4 | 4 | 4 | 4 | 4 | 4 | 4 | 4 | 3 | 4 | 43 |
| Mayo-Wilson et al.,[29] | 4 | 3 | 4 | 1 | 1 | 3 | 4 | 4 | 4 | 4 | 4 | 36 |
| Cembranel et al.,[30] | 3 | 4 | 4 | 4 | 2 | 1 | 3 | 3 | 3 | 3 | 1 | 31 |
| Low et al.,[31] | 3 | 4 | 4 | 3 | 4 | 4 | 1 | 4 | 4 | 1 | 1 | 33 |
| Pasricha et al.,[32] | 3 | 4 | 4 | 4 | 4 | 4 | 3 | 3 | 4 | 3 | 4 | 40 |
| Thompson et al.,[33] | 4 | 4 | 4 | 4 | 4 | 3 | 4 | 4 | 4 | 3 | 4 | 42 |
| Abdullah et al.,[34] | 3 | 4 | 4 | 3 | 4 | 4 | 3 | 3 | 3 | 3 | 3 | 37 |
| [De-Regil et al.,](file:///C:\Users\MAHE\Desktop\De-Regil%202011)[22] | 4 | 4 | 4 | 4 | 4 | 4 | 4 | 4 | 4 | 1 | 3 | 40 |
| Ramakrishnan et al.,[35] | 2 | 2 | 3 | 3 | 3 | 2 | 2 | 3 | 3 | 2 | 2 | 27 |
| Matsuyama et al.,[36] | 3 | 3 | 4 | 4 | 2 | 3 | 1 | 2 | 3 | 3 | 3 | 31 |
| De-Regil et al.,[23] | 4 | 4 | 4 | 4 | 4 | 4 | 4 | 4 | 4 | 4 | 4 | 44 |
| Aaron et al., ^37^ | 4 | 3 | 4 | 4 | 4 | 4 | 4 | 4 | 4 | 3 | 4 | 42 |
| Das et al.,[38] | 3 | 4 | 4 | 4 | 4 | 4 | 4 | 4 | 4 | 1 | 4 | 40 |
| Das et al.,[39] | 3 | 4 | 4 | 4 | 4 | 4 | 4 | 4 | 4 | 1 | 4 | 40 |
| Salam et al.,[40] | 3 | 4 | 3 | 4 | 4 | 4 | 4 | 4 | 4 | 1 | 4 | 39 |
| Eichler et al.,[41] | 4 | 4 | 3 | 3 | 2 | 3 | 3 | 2 | 3 | 1 | 2 | 30 |
| Gera et al.,[42] | 3 | 4 | 4 | 4 | 4 | 4 | 3 | 4 | 4 | 4 | 3 | 41 |
| De-Regil et al.,[24] | 4 | 4 | 4 | 4 | 4 | 4 | 4 | 4 | 4 | 1 | 4 | 41 |
| Best et al.,[43] | 3 | 3 | 3 | 3 | 4 | 4 | 1 | 1 | 1 | 1 | 4 | 28 |
| Dewey et al.,[44] | 3 | 3 | 3 | 3 | 3 | 3 | 1 | 2 | 3 | 3 | 4 | 31 |
| Kristjansson et al.,[25] | 4 | 4 | 4 | 4 | 4 | 4 | 4 | 4 | 3 | 4 | 4 | 43 |
| McDonagh et al.,[45] | 3 | 4 | 3 | 4 | 4 | 3 | 2 | 3 | 2 | 1 | 4 | 33 |
| Gera et al.,[46] | 3 | 1 | 3 | 4 | 3 | 4 | 3 | 3 | 4 | 4 | 3 | 35 |
| Gera et al.,[47] | 3 | 1 | 3 | 4 | 3 | 4 | 3 | 3 | 4 | 4 | 3 | 35 |
| Sun et al.,[48] | 3 | 4 | 4 | 4 | 3 | 4 | 2 | 2 | 4 | 3 | 3 | 36 |
| Taylor-Robinson et al., [26] | 4 | 4 | 4 | 4 | 4 | 4 | 4 | 4 | 3 | 3 | 4 | 42 |
| Girum and Wasie[49] | 3 | 2 | 4 | 2 | 3 | 3 | 1 | 1 | 4 | 2 | 3 | 28 |
| Huang et al.,[50] | 3 | 3 | 3 | 4 | 3 | 4 | 3 | 3 | 4 | 4 | 3 | 37 |
| Dangour et al.,[27] | 4 | 4 | 4 | 4 | 4 | 4 | 4 | 4 | 4 | 1 | 4 | 41 |
| Athuman et al.,[28] | 4 | 4 | 4 | 3 | 4 | 4 | 4 | 4 | 4 | 1 | 4 | 40 |
